# Brain lesion segmentation through image synthesis and outlier detection

**DOI:** 10.1016/j.nicl.2017.09.003

**Published:** 2017-09-08

**Authors:** Christopher Bowles, Chen Qin, Ricardo Guerrero, Roger Gunn, Alexander Hammers, David Alexander Dickie, Maria Valdés Hernández, Joanna Wardlaw, Daniel Rueckert

**Affiliations:** a Department of Computing, Imperial College London, UK; b Imanova Ltd., London, UK; c King's College London & Guy's and St Thomas' PET Centre, Division of Imaging Sciences and Biomedical Engineering, St Thomas' Hospital, King's College London, UK; d Department of Neuroimaging Sciences, University of Edinburgh, UK; e Department of Medicine, Imperial College London, UK

## Abstract

Cerebral small vessel disease (SVD) can manifest in a number of ways. Many of these result in hyperintense regions visible on *T*_2_-weighted magnetic resonance (MR) images. The automatic segmentation of these lesions has been the focus of many studies. However, previous methods tended to be limited to certain types of pathology, as a consequence of either restricting the search to the white matter, or by training on an individual pathology. Here we present an unsupervised abnormality detection method which is able to detect abnormally hyperintense regions on FLAIR regardless of the underlying pathology or location. The method uses a combination of image synthesis, Gaussian mixture models and one class support vector machines, and needs only be trained on healthy tissue. We evaluate our method by comparing segmentation results from 127 subjects with SVD with three established methods and report significantly superior performance across a number of metrics.

## Highlights

• A novel method for image synthesis is presented
• The method is particularly suited to synthesising non-pathological brain images
• Synthetic subject specific non-pathological images are used to segment hyperintense lesions on 127 magnetic resonance images
• Resulting segmentations compare favourably to existing methods
• A particular improvement is observed in the segmentation of smaller lesions, stroke lesions and lesions closer to the cortex

## 1 Introduction

**Fig. 1:**
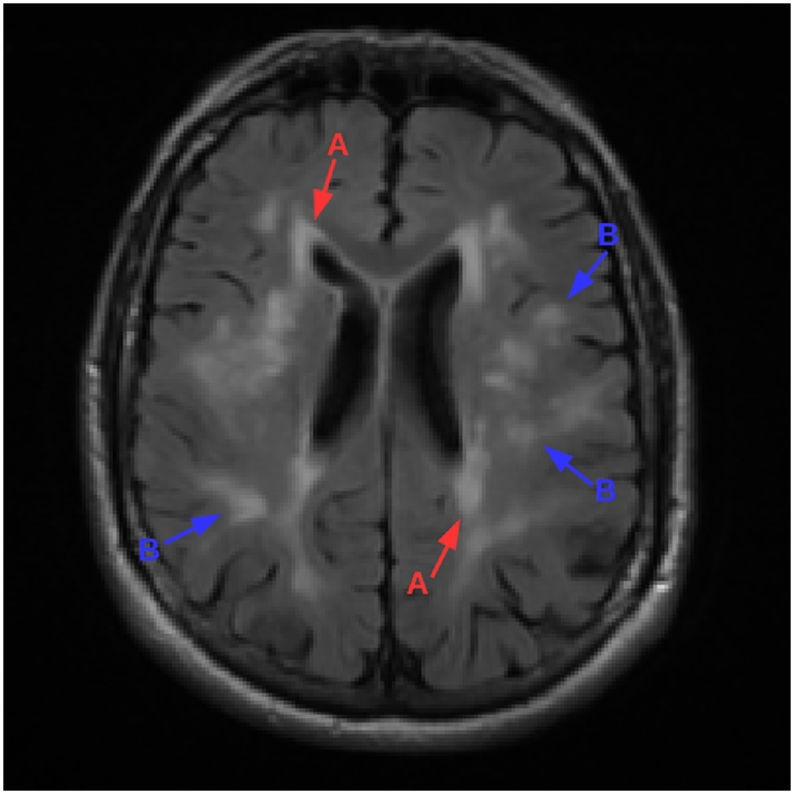
*T*_1_-w (left) and FLAIR (right) image of a subject with periventricular (labeled A) and deep (labeled B) white matter lesions. Note that pathology is more visible on the FLAIR image than it is on the *T*_1_-w image.

Cerebral small vessel disease (SVD) is common in the elderly with severe cases leading to cognitive impairment and dementia. Whilst the aetiology of SVD is not always clear, risk factors include age, smoking, and elevated blood pressure (van Dijk et al., 2008). SVD can manifest in a number of ways (Wardlaw et al., 2013), usually as a result of intrinsic brain small vessel abnormality leading to an inadequate blood supply (ischemia). Brain tissue damaged as a result of ischemia presents as hyperintense on *T*_2_-weighted (*T*_2_-w) magnetic resonance (MR) images and often hypointense on *T*_1_-weighted (*T*_1_-w) images, see Fig. 1.

SVD can also lead to lacunes (fluid filled cavities  <20 mm diameter with MR signal properties similar to cerebrospinal fluid (CSF), sometimes with a *T*_2_-w hyperintense ring); enlarged perivascular spaces (extracerebral fluid around vessels,  <2 mm diameter, similar MR appearance to small lacunes without *T*_2_-w hyperintense ring); and cerebral microbleeds (leakage of blood cells into perivascular tissue, visible as  <10 mm diameter hypointensity on *T*_2_ *-weighted and susceptibility weighted MR sequences) (Wardlaw et al., 2013).

Most attempts to automatically quantify SVD (Caligiuri et al., 2015) have focused on the accurate segmentation of hyperintense lesions within the white matter (WM) on fluid attenuated inversion recovery (FLAIR) MR images (Hajnal et al., 1992). FLAIR is the most useful MR sequence for the detection of these lesions as it is a *T*_2_-w sequence in which signals from confounding sources of hyperintensity, primarily CSF, are canceled out. There has been comparatively little work on identifying the other manifestations of SVD such as lacunes (Ghafoorian et al., 2016), perivascular spaces (Valdés Hernández et al., 2013b) and microbleeds (Kuijf et al., 2012).

Of the proposed methods to segment WM lesions, very few are publicly available. Of these, the most common comparator methods belong to the Lesion Segmentation Toolbox^1^ (LST). The LST contains two methods, the Lesion Growth Algorithm (LGA) (Schmidt et al., 2012) and Lesion Prediction Algorithm (LPA). Both methods were developed for the segmentation of multiple sclerosis (MS) lesions. However due to the similarities between the appearance of Multiple Sclerosis (MS) lesions and WM lesions, MS lesion segmentation algorithms García-Lorenzo et al., 2013, Lladó et al., 2012 and WM lesion segmentation algorithms can often be used interchangeably. As such, both methods from the LST are commonly used as benchmarks for hyperintense lesion segmentation. Another publicly available method is LesionTOADS (Shiee et al., 2010), which simultaneously performs both tissue and lesion segmentation in an unsupervised manor. At the moment, LPA is the closest the field has to a readily available and robust gold standard, having been shown to consistently offer good results across a number of datasets despite being primarily an MS lesions segmentation tool. However, a recently published method, BIANCA (Griffanti et al., 2016), reports some promising results surpassing LPA in a number of metrics including Dice Similarity Coefficient (DSC) (BIANCA: 0.79, LPA: 0.76) on a neurodegenerative dataset (*n* = 85).

Image synthesis is the name given to the process of synthesising an image from a particular modality from images from one or more other modalities. The majority of existing methods (Roy et al., 2010a,Roy et al., 2010b, Roy et al., 2011; Konukoglu et al., 2013, Rueda et al., 2013, Huang and Wang, 2013, Cao et al., 2014, Ye et al., 2013, Tsunoda et al., 2014, Cao et al., 2013, Roy et al., 2014, Huang et al., 2016, Roy et al., 2016 for image synthesis stem from an initial framework proposed in Hertzmann et al. (2001), whereby a dictionary of source/target patch pairs is initially learned, with synthesis being performed on a patch by patch basis by finding the closest source patch and propagating the corresponding target patch to the synthetic image. The main sources of variation in the methods based upon this being in differing techniques to efficiently search the large patch dictionary, and additional constraints to ensure the selected patch is spatially coherent with its neighbours. Another family of approaches treats the problem as one of regression and looks to learn a set of functions which will map an intensity from one modality to another either through regression forests Jog et al., 2013, Alexander et al., 2014, Jog et al., 2015, Jog et al., 2017 or by learning the most common intensity relationships (Kroon and Slump, 2009). Recently, deep learning solutions have been also been proposed Van Nguyen et al., 2015, Vemulapalli et al., 2015, Sevetlidis et al., 2016 demonstrating some good results. Further approaches include the use of deformable atlases (Miller et al., 1993), registration and intensity fusion (Burgos et al., 2013) and generative models (Cardoso et al., 2015). The ability of the latter to identify white matter lesions as outliers was also explored.

The majority of these approaches aim to address the problem of multi-modal registration Iglesias et al., 2013, Cao et al., 2013, Roy et al., 2014, Cao et al., 2014, Kroon and Slump, 2009, Jog et al., 2013, Alexander et al., 2014, Chen et al., 2015b or super resolution (Roy et al., 2010a,Roy et al., 2010b, Roy et al., 2011, Roy et al., 2013, Zhang et al., 2012, Konukoglu et al., 2013, Rueda et al., 2013, Alexander et al., 2014. The idea of “pseudo-healthy” image synthesis has also been explored whereby the aim is to synthesise a pathology free subject specific image in a target modality. This has been used by Ye et al. (2013) to perform tumour segmentation, by Tsunoda et al. (2014) to detect lung nodules on CT images, and had its potential for WM lesion segmentation suggested in passing by Roy et al. (2013). This approach is most useful when pathology is not visible on one modality, but visible on another. Synthesising a pathology free version of the pathological modality allows abnormalities to be identified though subtraction. This is not necessarily the case in SVD where pathology can be visible on both *T*_1_-w and FLAIR images (Fig. 1). In fact, existing methods have been demonstrated to synthesise hyperintensities Roy et al., 2013, Jog et al., 2017, and even exploit this (Jog et al., 2015) for the purposes of lesion segmentation in the absence of FLAIR. However careful design of synthesis algorithm allows a pathology free FLAIR to be synthesised in the presence of *T*_1_-w visible pathology.

Here we build upon our previous work (Bowles et al., 2016) and present a method for FLAIR hyperintensity segmentation though image synthesis and outlier detection. We first describe a method for robust “pseudo-healthy” image synthesis in the presence of *T*_1_-w visible pathology using kernel regression to learn the expected relationships between *T*_1_-w and FLAIR intensities at each location within the brain. Subtraction of the “pseudo-healthy” image from the acquired image then gives an indication of pathology. A Gaussian mixture model is then used to locate abnormally bright areas in the FLAIR image. These two pieces of information are then combined with an SVD atlas within a one class classification framework, and the output is post-processed using a conditional random field (CRF).

The proposed method is unsupervised in the sense that it does not require any manually segmented ground truth images to train on, and is therefore less prone to overfitting than supervised methods. It is also flexible enough to segment a wide range of abnormalities without needing to be trained on examples of different pathologies. It does however need to be trained on non-pathological tissue. This can either be from images of healthy subjects, or from the regions outside of manual segmentations of pathological images.

### 1.1 A note on terminology

**Fig. 2:**
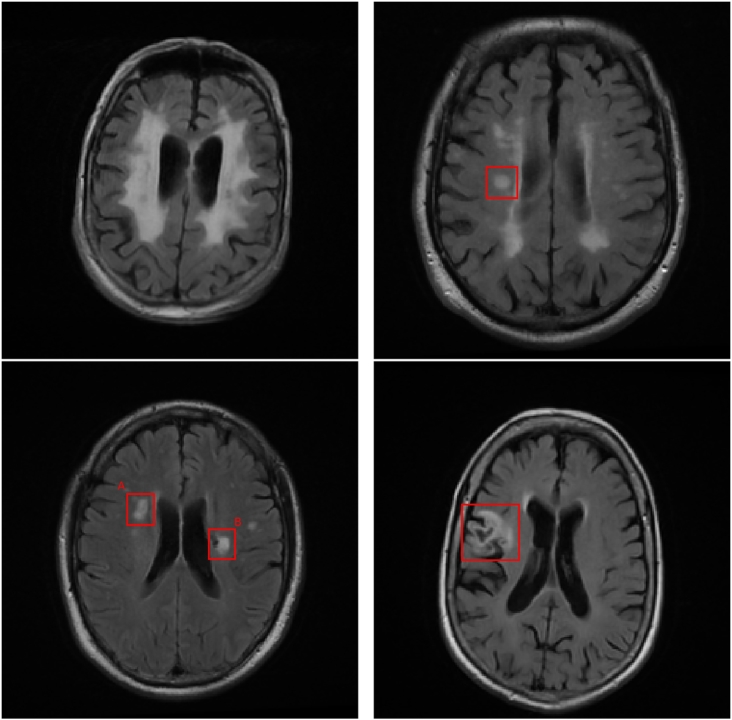
Examples of different hyperintensities relating to SVD. Top left: White matter hyperintensity of presumed vascular origin. Top right: Recent small subcortical infarct. Bottom left: A: Evolution of a recent small subcortical infarct into a *T*_2_-w hyperintensity, B: Lacunar cavity forming at the edge of a WMH of unclear origin. Bottom right: Cortical infarct.

The terminology and definitions surrounding SVD and associated imaging features can vary significantly between studies (Wardlaw et al., 2013). To avoid confusion we define the following relevant terms explicitly in line with those given by Wardlaw et al. with examples of each shown in Fig. 2. The term white matter hyperintensities of presumed vascular origin (WMH_pvo_) refers to the lesions within the WM which appear hyperintense on *T*_2_-w MRI (including FLAIR) which are often present in images of older people. WMH_pvo_ are often symmetrical and their aetiology is unclear. The term recent small subcortical infarct (RSSI) refers to a *T*_2_-w/DWI hyperintense region indicating a recent infarction. An RSSI will evolve into either a lacunar cavity (*T*_1_-w/*T*_2_-w hypointense “space”, usually with a *T*_2_-w hyperintense ring) or *T*_2_-w hyperintensity. We use the term white matter hyperintensity (WMH) to include *T*_2_-w hyperintensities caused by WMH_pvo_, RSSIs, RSSIs which have evolved into *T*_2_-w hyperintensity and the *T*_2_-w hyperintense areas around lacunar cavities. Finally, we use the term cortical infarct to refer to *T*_2_-w hyperintense regions which appear wholly or partly in the cortical grey mater (GM) following an arterial distribution.

Whilst MS lesions also appear as hyperintense WM on FLAIR (Polman et al., 2011), no MS pathology is present in any of our experiments, hence we reserve the definition of WMH to the above and refer to MS induced hyperintensities separately as MS lesions. No other cause of *T*_2_-w hyperintensity (e.g. cancer, traumatic brain injury) is discussed in this paper, or present in any experiments.

## 2 Method

### 2.1 Overview

The proposed method treats the problem of lesion segmentation as an outlier detection task. The first stage is to produce two likelihood maps:

**L**^**SYN**^, is formed by synthesising a healthy looking FLAIR image from a subject's *T*_1_-w image. Subtraction of this synthetic FLAIR image from the subject's true FLAIR image produces a difference image which represents the likelihood of a FLAIR voxel intensity to be abnormal, given the subject's *T*_1_-w image and an expected pre-determined relationship between healthy *T*_1_-w and FLAIR intensities. This value is low in the presence of healthy tissue, and high in the presence of pathological tissue.

**L**^**FLAIR**^, represents the likelihood for a given FLAIR voxel to be abnormal given a pre-computed Gaussian-mixture model of expected FLAIR intensities at that location.

These likelihood maps are then combined with a WMH_pvo_ probability atlas within a one-class classification framework to provide a single likelihood map reflecting the degree of abnormality at each voxel. Finally, a conditional random field (CRF) is applied, resulting in a binary segmentation.

**L**^**SYN**^, **L**^**FLAIR**^, and the one-class classifier used to combine them all require a training set of healthy subjects. **L**^**SYN**^ a requires both *T*_1_-w and FLAIR images, whilst **L**^**FLAIR**^ and the one-class classifier require FLAIR images. There is no requirement for the three training sets to include the same subjects, however it is practical to use the same set of FLAIR images. We therefore refer to the *T*_1_-w and FLAIR images in this dataset as **T**^**train**^ and **F**^**train**^ respectively.

### 2.2 Preprocessing

Preprocessing is required to normalise the images to a standard set of properties, ensuring subsequent steps are robust to the heterogeneous image characteristics found both within and between medical imaging datasets. These preprocessing steps also compute a number of segmentations and transformations which are required in subsequent steps. Preprocessing is identical for both the training set and the images we wish to segment, which we refer to from here as the test set.

#### 2.2.1 Registration

Registration is performed using the MIRTK suite of registration tools (available at^2^). A rigid transformation from the *T*_1_-w to FLAIR image spaces is first computed. A free-form deformation (FFD) (Rueckert et al., 1999) transformation (Resolution levels: 40 mm, 20 mm, 10 mm, 5 mm; Image dissimilarity measure = SSD; Bending energy weight = 0.1) is then computed between the *T*_1_-w image in FLAIR image space and an MNI template (ICBM 2009a Nonlinear Symmetric, available at^3^). The inverse transformation is also computed.

#### 2.2.2 Bias correction, brain extraction and anatomical segmentation

A multi-atlas based anatomical segmentation tool, MALPEM (Ledig et al., 2015) (available at^4^), is applied to the *T*_1_-w image providing both binary and probabilistic segmentations of 142 anatomical structures. As part of the segmentation process, MALPEM applies bias field correction using the N4 (Tustison et al., 2010) algorithm and brain extraction using the *pincram* algorithm (Heckemann et al., 2015), outputting the resulting *T*_1_-w image and brain mask. WM and GM probability maps are computed from the probabilistic segmentations.

Bias correction is performed separately on the FLAIR image using the N4 algorithm and the *T*_1_-w brain mask is transformed to FLAIR image space, re-sampled using nearest-neighbour interpolation and used to crop the FLAIR image.

#### 2.2.3 Intensity normalisation

Intensity normalisation is an especially important procedure since many subsequent steps involve direct comparisons between voxel intensities across images from different subjects. However, the nature of hyperintense lesions means that several commonly used normalisation methods are inadequate. The often used approach of linear scaling of intensities to the range [0,1] with a certain percentage of the lowest and highest intensities saturated at 0 and 1 respectively (Cao et al., 2014) will result in different intensity mappings dependent on the volume of hyperintense lesions compared to the percentage of voxels saturated. Histogram matching (Ye et al., 2013) suffers similar problems in the presence of hyperintensities. Scaling images to have a zero mean and unit variance (Hertzmann et al., 2001) is also inadequate as the degree of hyperintensity will bias both the mean and the variance of the image.

To make intensity normalisation invariant to degree of hyperintensity and atrophy common in elderly subjects, we employ the method used in Huppertz et al. (2011). Two sets of voxels corresponding to WM and GM are produced by filtering probabilistic WM and GM masks to include only voxels with a  >95% probability of being of that tissue class. Next, these two sets are further refined by intensity to contain only intensities which fall within a 95% confidence interval so as to remove outliers. This leaves two sets which are highly likely to contain WM and GM, and which are not outliers within these groups, therefore corresponding only to healthy tissue. The mean of each set of intensities is calculated to give the expected intensity of healthy tissue in the WM and GM. The mean of these two values is subsequently calculated to provide a single fixed point. Finally, image intensities are scaled linearly such that this fixed point is set to the arbitrary value of 1000.

This method is applied to both the *T*_1_-w image and FLAIR image, using the probabilistic WM and GM masks derived from the previously computed anatomical segmentations. In the case of the FLAIR image these masks are transformed to FLAIR image space and re-sampled using linear interpolation.

### 2.3 Training

In order to produce **L**^**SYN**^ and **L**^**FLAIR**^, two sets of models are trained. The first is a synthesis model that learns the relationship between *T*_1_-w and FLAIR intensities. The second is a Gaussian mixture model (GMM) which learns the expected intensity distributions within a FLAIR image.

**Fig. 3:**
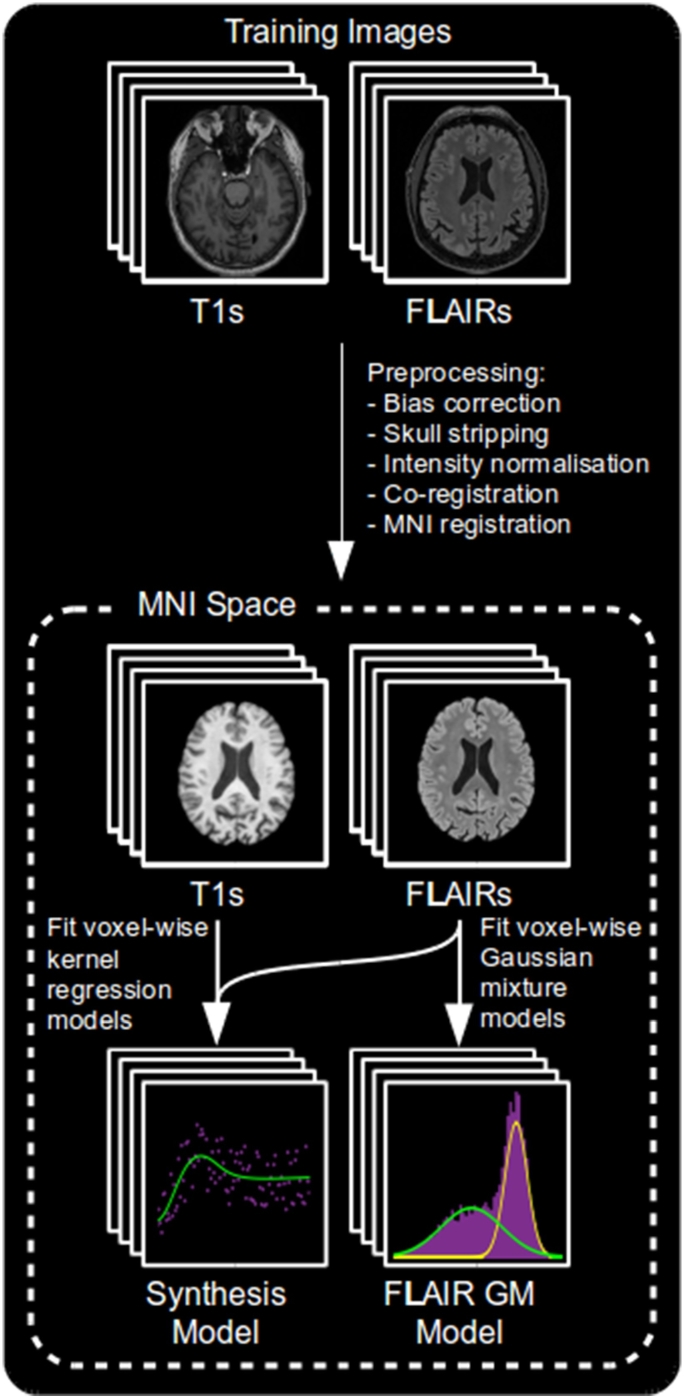
An overview of the training process.

To account for imperfect tissue segmentation, common in the presence of hyperintense lesions, and for intensity variations within a tissue type, we compute both sets of models in a voxel-wise manner within MNI space. A separate model is produced for each voxel, computed using information taken from a patch around that voxel in each co-registered training image. The process of training both models is summarised in Fig. 3.

#### 2.3.1 Synthesis model

The key step for the computation of **L**^**SYN**^ is the calculation of a pseudo-healthy FLAIR image from a subject's *T*_1_-w image. Our proposed method uses voxel-wise kernel regression to learn a direct mapping between healthy *T*_1_-w and FLAIR intensities at each voxel.

A set of *n* training image pairs **T**^**train**^ and **F**^**train**^ are transformed to MNI space using the transformations calculated during preprocessing and re-sampled onto a 1 mm isotropic voxel lattice. Intensities in **T**^**train**^ are capped at a value *t*_max_. At each voxel **x**, two one-dimensional vectors **t**_**x**_ and **f**_**x**_ are formed from **T**^**train**^ and **F**^**train**^ respectively containing the voxel intensities from an *a*-by-*a*-by-*a* patch around **x** in each image. A kernel regression model **M**_**x**_ with bandwidth *h* is computed relating **t**_**x**_ to **f**_**x**_ and evaluated at *m* equally spaced values *k* between 0 and *t*_max_.

(1) $$M_{x}(k)=\frac{\sum_{i}(K((t_{x}(i))/h)f_{x}(i))}{\sum_{i}K((k-t_{x}(i))/h)},K(p)=\frac{1}{\sqrt{2\pi }}e^{-\frac{1}{2}p^{2}}.$$

Higher values of *m* and *t*_max_ result in more accurate synthesis at the cost of model size and computation time, whilst the number of voxels (*na*^3^) must be sufficiently large to contain enough information to fit the model. Preliminary experiments showed that *m* = 100, *t*_max_ = 1500, *n* = 20 and *a* = 5 were sufficient to produce useful images whilst remaining tractable ( <4 h to train,  <1 s to synthesise), with larger values having negligible impact on final results.

**Fig. 4:**
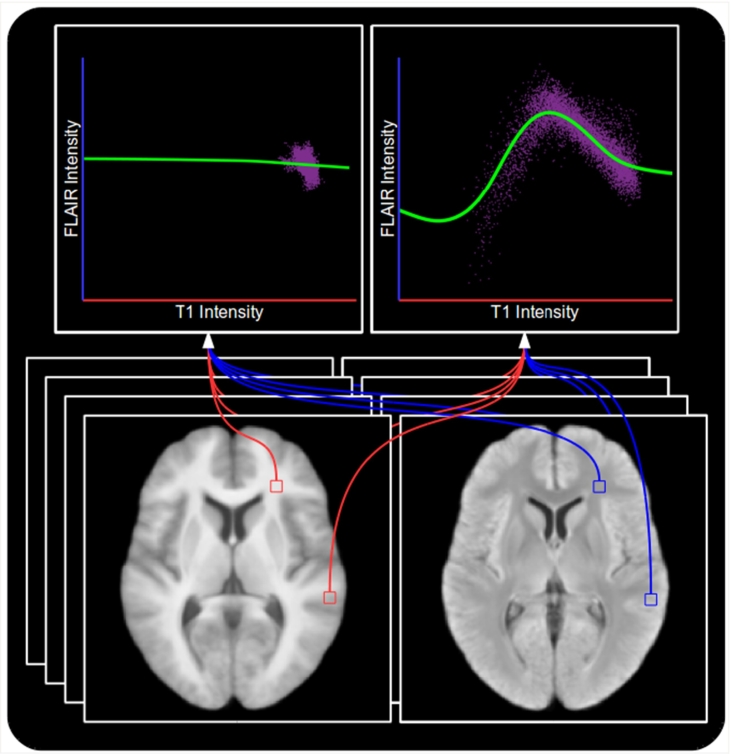
Two models produced using kernel regression to act as a mapping from *T*_1_-w to FLAIR intensities. Top left: A model produced at a location within the WM which contains only WM voxels. Top right: A model produced at a location which can contain WM, GM or CSF voxels. Bottom left: Mean *T*_1_-w training image. Bottom right: Mean FLAIR training image. Note that the model produced from WM, GM and CSF voxels is more complex than the one produced within the WM as a result of having to capture more intensity relationships, and that the extrapolation in the case of the latter provides the ability for the model to predict healthy WM FLAIR intensities even in the presence of *T*_1_-w visible pathology.

An example showing the models produced at two voxels is shown in Fig. 4. The top right figure clearly displays the desired relationships in a location which can contain WM, GM or CSF. The brightest *T*_1_-w intensities correspond to darker FLAIR intensities, corresponding to WM appearing brighter on *T*_1_-w images than on FLAIR. GM appears darker on *T*_1_-w images and brighter on FLAIR, explaining the peak of the model. Finally, the darkest *T*_1_-w intensities correspond to CSF, as is the case on FLAIR, which is represented by the leftmost section of the model. However, in the top left figure we have the model formed in a location containing only WM, equivalent to the rightmost section of the previous model. Since there is no more information upon which to fit the model, the model extrapolates to predict the same FLAIR intensity across the whole range of *T*_1_-w intensities. This gives the model the desired ability to predict normal looking WM even in the presence of hypo-intense *T*_1_-w visible lesions, such as those in Fig. 1.

**Fig. 5:**
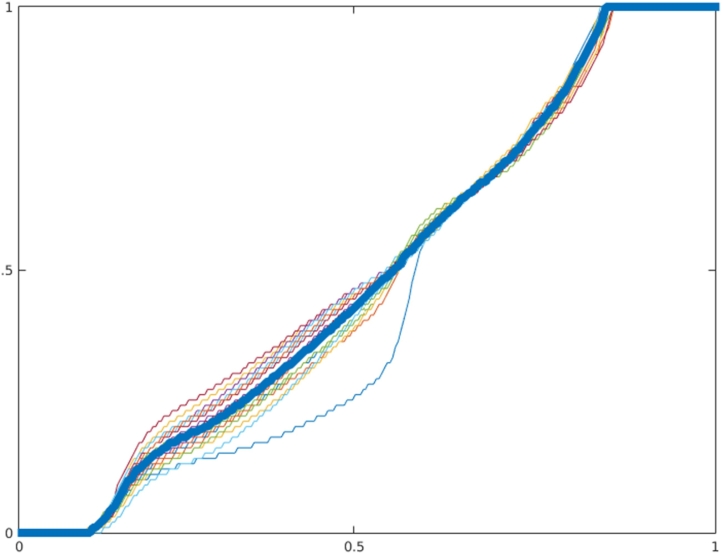
Transfer functions computed to map synthetic FLAIR images to their corresponding training FLAIR images. Thick blue line indicates the median which is used to correct all images.

**Fig. 6:**
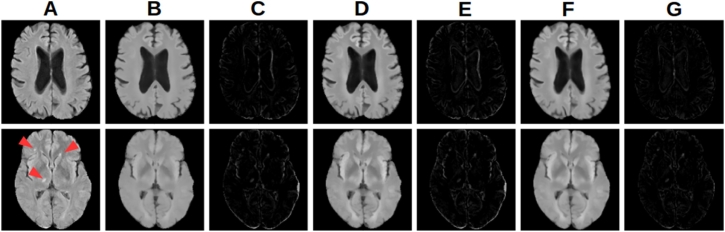
Effects of intensity correction and registration of synthetic images on a (top) pathology free and (bottom) pathological subject. (A) FLAIR image. (B) Rigidly registered synthetic image. (C) Difference image from (A) to (B). (D) Rigidly registered intensity corrected synthetic image. (E) Difference image from (A) to (D). (F) FFD registered intensity corrected synthetic image. (G) Difference image from (A) to (F). Note that the intensity correction and FFD registration do not prevent detection of the pathology (arrows).

A consequence of using kernel regression for synthesis is that the contrast between WM and GM in the synthetic image is reduced. This is due to the smoothing effect encouraging the model away from the extreme intensity values and towards the mean. As a result, the very highest and lowest FLAIR intensities would never be synthesised. To correct this, an intensity transfer function is computed for each subject in **T**^**train**^ by using histogram matching to match the intensity histograms of the synthesised image to the FLAIR image. The median of these transfer functions (Fig. 5) is computed and used to correct all images, the effects of which can be seen in Fig. 6.

#### 2.3.2 Gaussian mixture model

**Fig. 7:**
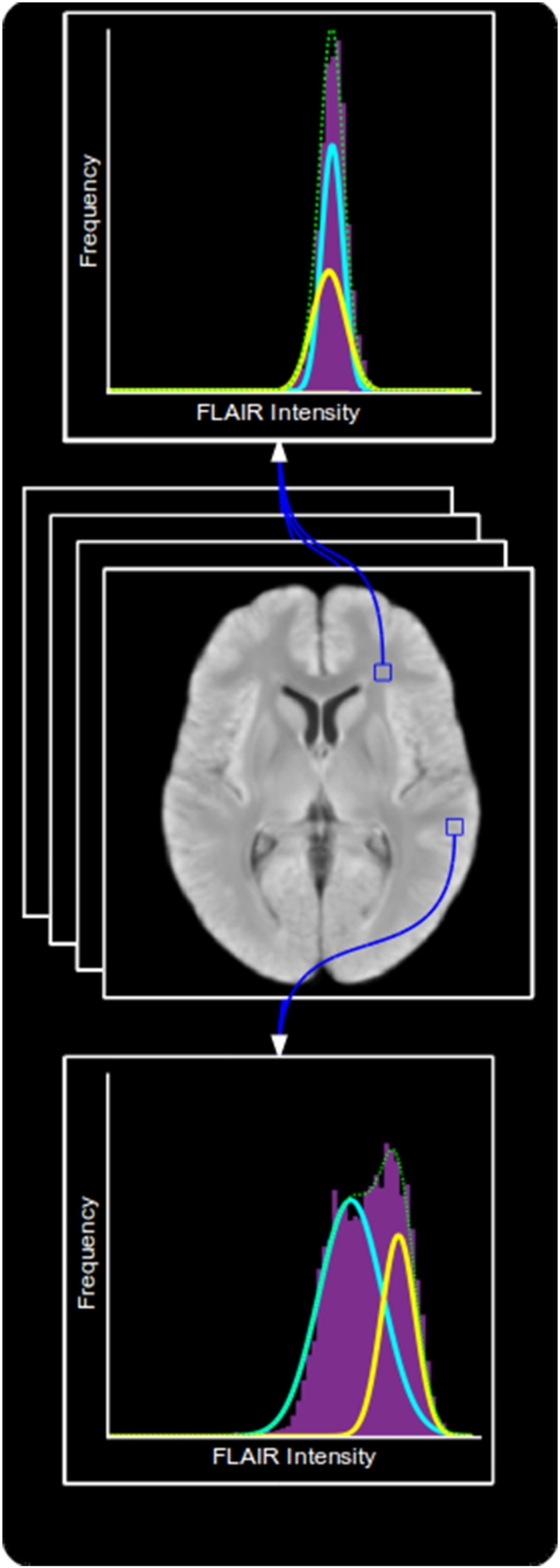
Two GMMs learned to represent the normal distribution of FLAIR intensities around their corresponding voxel. Top: A model produced at a location near the boarder between GM and WM. Middle: Mean FLAIR training image. Bottom: A model produced at a location within the WM. Note that the model produced from the border between WM and GM has two distinct components representing the two tissue types, whereas the model produced from within the WM contains two very similar components.

**L**^**FLAIR**^ is a representation of the likelihood of a voxel intensity being abnormal given previous knowledge of the expected distribution of intensities at each location. The distribution of intensities found across the whole brain is wide and complex, however at a voxel level, these distributions become narrower and easier to represent. It is common to treat intensities within a single tissue class as belonging to a Gaussian distribution, hence why many tissue segmentation algorithms are based upon an Expectation Maximisation (EM) framework (Zhang et al., 2001). Intensities at a single voxel across a number of co-registered images will therefore likely belong to either one (when the voxel lies within a tissue class) or a mixture of two (when the voxel lies on the boundary between tissue classes) Gaussian distributions. We therefore use an EM approach (McLachlan and Peel, 2000) to learn a GMM with two components from **F**^**train**^ at each voxel in MNI space. Due to a limited number of training images and the need for a lot of samples to confidently fit the GMM, voxels in a *b*-by-*b*-by-*b* patch around the target voxel are used, whilst boundary cases are handled by only considering non-zero intensities. Preliminary experiments showed that *b* = 5 provided sufficient information to confidently fit the models with 20 training images. An example showing the models produced at the same two locations as shown in Fig. 4 is shown in Fig. 7.

### 2.4 Testing

**Fig. 8:**
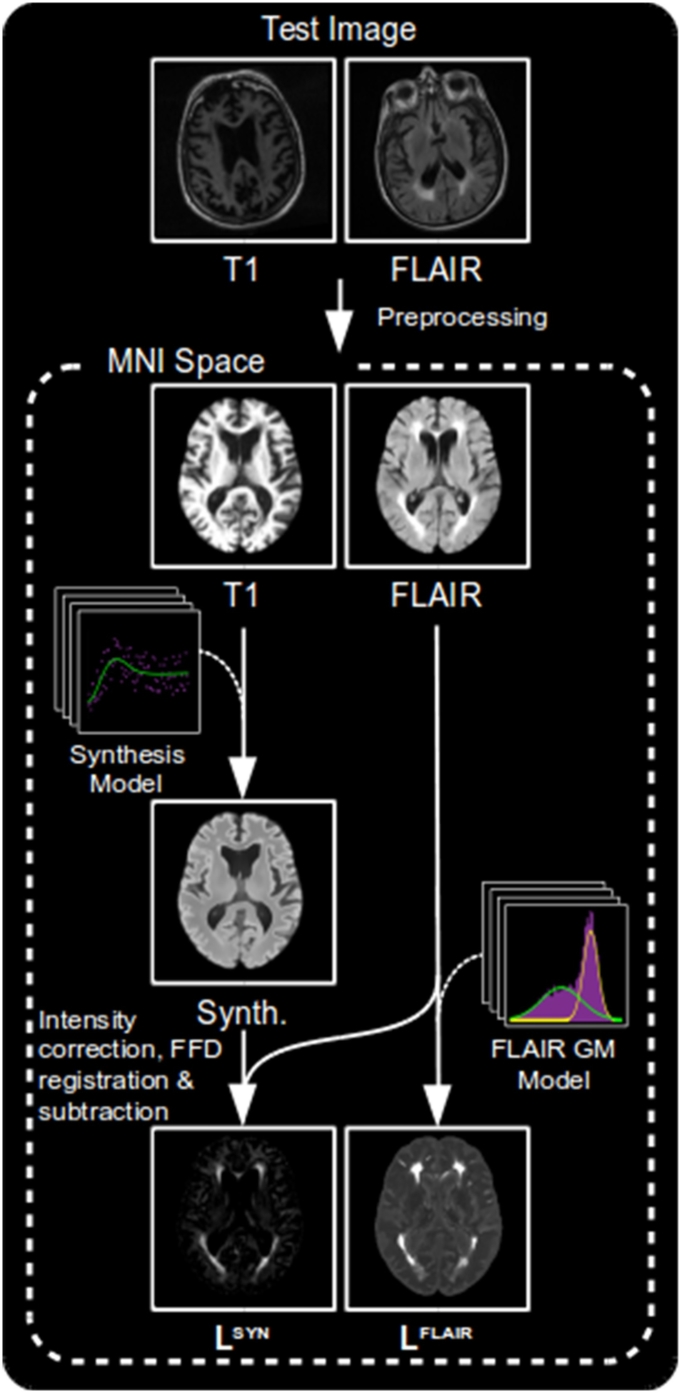
An overview of the process of creating the **L**^**SYN**^ and **L**^**FLAIR**^ likelihood maps.

Having produced the two sets of models, we can now apply them to test images to produce **L**^**SYN**^ and **L**^**FLAIR**^. A summary of the process can be seen in Fig. 8.

#### 2.4.1 **L**^**SYN**^

**Fig. 10:**
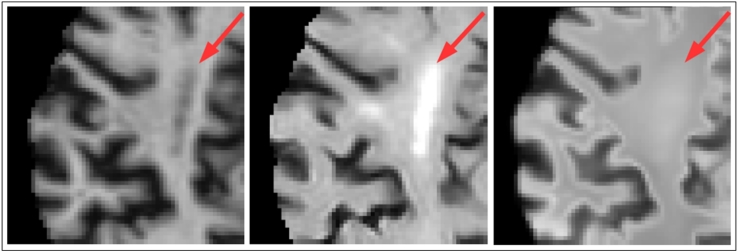
A case where a lesion is correctly synthesised as the same intensity as the surrounding WM. Left: *T*_1_-w image. Middle: FLAIR image. Right: Corresponding synthetic FLAIR image.

To synthesise a voxel **x** of synthetic image **S** using regression model *M*, the corresponding voxel in the subject's *T*_1_-w image, **t**_**x**_, is capped at *t*_max_ and turned into an index *i* = ⌈*m* **t**_**x**_/*t*_max_⌉. This index is then used to index into **M**_**x**_ to give **S**_**x**_. The intensities of **S** are finally adjusted using the previously computed transfer function. An example of successful pseudo-healthy synthesis in the presence of WMH can be seen in Fig. 10.

As we will be performing voxel-wise comparisons of **F** and **S**, it is important that we have a good registration between them. As discussed earlier, studies have shown the benefits of using synthetic images to achieve more accurate multi-modal registrations by reducing the problem to a mono-modal one between the synthetic and target images. We therefore register **S** directly to **F**, producing **S**^**F**^. Despite this registration theoretically being rigid, we introduce a small non-linear term. This is to make the registration more robust to artefacts present in either one of the images, in particular distortions caused by eddy currents, and by partial volume effects often caused by FLAIR images having a large slice thickness.

**Fig. 9:**
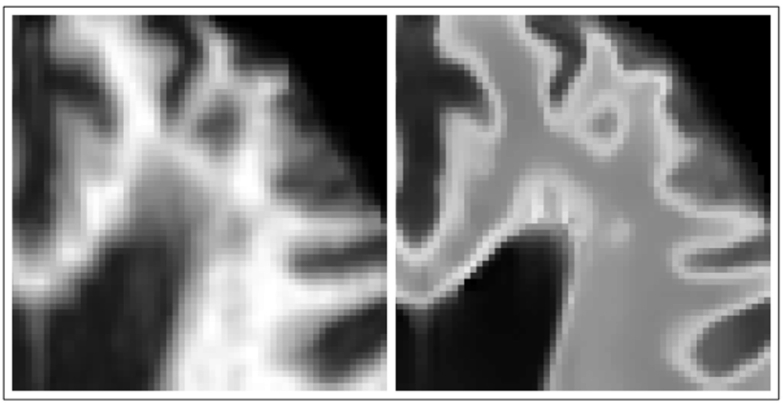
An example where periventricular WMH has been synthesised. Left: Normalised *T*_1_-w image. Right: Corresponding synthetic FLAIR image.

We must make a special case for the region around the ventricles. Small hyper-intensities around the ventricular wall known as “bands” and “caps” are common in aging and can be a result of a several phenomena (Barkhof et al., 2011). The presence of these “bands” and “caps” in the otherwise healthy training data leads to the undesired synthesis of clinically relevant WMH around the ventricles, see Fig. 9. To avoid this leading to inaccurate segmentations, the intensities of WM in the synthetic images within 15 mm of the ventricles, as determined by a distance transform, are capped at a value corresponding to the expected intensity of healthy WM in this region.

**Fig. 11:**
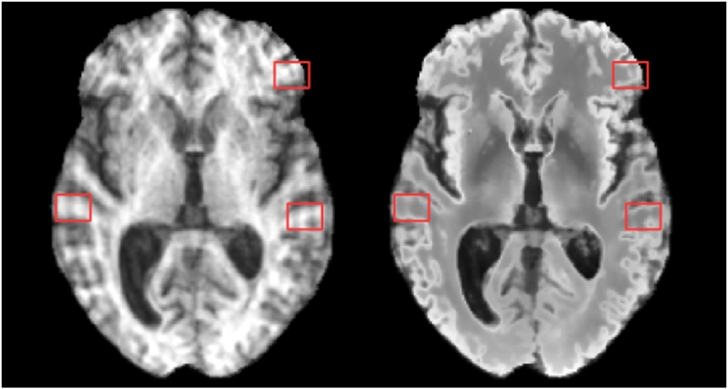
A case where ringing artefacts in a subject's *T*_1_-w image results in errors in the synthesised FLAIR image whereby juxtacortical WM is synthesised as GM in the indicated locations. Left: *T*_1_-w image. Right: Corresponding synthetic healthy FLAIR image.

**Fig. 12:**
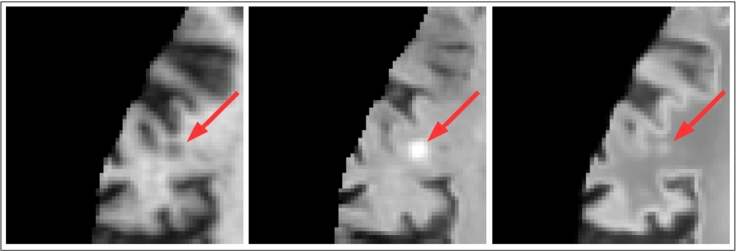
A case where a lesion close to the cortex is mistakenly synthesised as hyper-intense. Left: *T*_1_-w image. Middle: FLAIR image. Right: Corresponding synthetic FLAIR image.

**L**^**SYN**^ is then computed as **F** −**S**^**F**^. At this point an approximate segmentation could be formed by applying a threshold to **L**^**SYN**^, however there are situations which could cause errors to arise in the resulting segmentation. Artefacts in the *T*_1_-w image, particularly ringing artefacts, will cause errors in the synthesised image. These could introduce both false positives (seen in Fig. 11), and false negatives should the ringing negate the signal from a lesion. Cortical infarcts can sometimes be synthesised as hyper-intense as a result of being treated like GM due to their proximity to the cortex, seen in Fig. 12. Whilst juxtacortical infarcts are brighter than normal GM on *T*_2_-w images, the difference in intensity will be small, and could fall under a threshold. Finally, the high slice thickness common in FLAIR images can result in partial volume effects. These are particularly visible in the axial plane at the boundaries between CSF and WM or GM, such as at the top of the 3rd and 4th ventricles and the base of the frontal and temporal lobes. The synthetic image formed from the higher resolution *T*_1_-w image will not suffer these effects and will therefore appear brighter within the brain matter, leading to potential false positives.

In order to limit false positives due to *T*_1_-w artefacts and FLAIR partial volumes, and to reinforce areas of small differences in **L**^**SYN**^ such as could be seen in the case of lesions in or near the cortex, additional information related to the brightness of the FLAIR image is required. We obtain this from **L**^**FLAIR**^.

#### 2.4.2 **L**^**FLAIR**^

To compute **L**^**FLAIR**^, a relative likelihood is computed at each voxel reflecting the likelihood of that voxel being abnormal given the previously computed GMMs. To assign a likelihood to a given voxel, **x** in a test image, the log-likelihood of the intensity of the voxel is computed using the corresponding two-component GMM, parametrised by weights (*w*_1,**x**_,*w*_1,**x**_), means (*μ*_1,**x**_,*μ*_2,**x**_) and standard deviations (*σ*_1,**x**_,*σ*_2,**x**_). The resulting value will be large for both abnormally hyper- and hypo-intense voxels. To ensure only hyper-intense voxels are identified the likelihood is set to zero in regions with a FLAIR intensity **f**_**x**_ less than the mean of the average intensities of GM and WM, previously set to 1000 during normalisation.

(2) $$L_{x}^{FLAIR}=\left\{\begin{array}{ll} w_{1,x}\frac{1}{\sqrt{2\sigma_{1,x}^{2}\pi }}e^{\frac{f_{x}-\mu_{1,x}^{2}}{2\sigma_{1,x}^{2}}}+w_{2,x}\frac{1}{\sqrt{2\sigma_{2,x}^{2}\pi }}e^{\frac{f_{x}-\mu_{2,x}^{2}}{2\sigma_{2,x}^{2}}}\; & \text{if}\;f_{x}\geq 1000\\ 0\; & \text{otherwise} \end{array}\right)$$

### 2.5 Combining **L**^**SYN**^ and **L**^**FLAIR**^

To combine the information from **L**^**SYN**^ and **L**^**FLAIR**^ we use a similar framework to that proposed in Karpate et al. (2015), where the authors combine a number of probability maps using a supervised SVM. We choose to use unsupervised one-class SVMs, such as in El Azami et al. (2016), to remove need for labeled data and to maintain the proposed method's flexibility by allowing it to be used for general abnormality detection and not be restricted to a particular pathology present in a training set.

#### 2.5.1 Training

The SVMs are trained using the same subjects which formed the training set used to train the models used to produce **L**^**SYN**^ and **L**^**FLAIR**^, with both likelihood maps in MNI space. A 3-by-1 feature vector is computed for each voxel containing the values of **L**^**SYN**^, **L**^**FLAIR**^ and an in house probabilistic WMH_pvo_ atlas generated by averaging co-registered manual WMH_pvo_ segmentations, a full description of which can be found in Chen et al. (2015a).

A separate one-class SVM is trained for WM and GM. Fifty-thousand training points are randomly sampled from the feature vectors coming from each tissue class with an outlier percentage of 5% and 0.3% for the WM and GM classifiers respectively. These percentages were chosen empirically by visually assessing the resulting classifier's tendency to over/under-segment within each tissue class. Apparent over-segmentation lead to the outlier percentage being increased, whilst under-segmentation lead to a decrease.

#### 2.5.2 Testing

To analyse a test image, the corresponding **L**^**SYN**^ and **L**^**FLAIR**^ likelihood maps are combined with the WMH_pvo_ atlas to form a feature vector at each voxel. Vectors are then classified using the previously trained one-class SVM corresponding to the tissue type which has the greater probability at that voxel. If the voxel falls outside of the decision boundary, and is therefore considered an outlier, a score is formed for that vector defined by its distance from the decision boundary. A single likelihood map, **L**^**SVM**^, is formed from these scores.

#### 2.5.3 CRF refinement

To binarize and remove false positives from **L**^**SVM**^ we apply a final post-processing step using a 3D fully connected CRF, described first in Krähenbühl and Koltun (2011) and extended to 3D and implemented in Kamnitsas et al. (2016).

## 3 Experiments

To evaluate the performance of the proposed method we compare it to three of publicly available methods for lesion segmentation. Two methods from the LST (available at^5^) LGA and LPA, and LesionTOADS (available at^6^).

### 3.1 Data

The data for our evaluation comes from a heterogeneous dataset containing data acquired using three different acquisition protocols. All image data were acquired at the Brain Research Imaging Centre of Edinburgh^7^ on a GE Signa Horizon HDx 1.5 T clinical scanner (General Electric, Milwaukee, WI), equipped with a self-shielding gradient set and manufacturer-supplied eight-channel phased-array head coil. Details of the protocols used for acquiring the data are given in Table 1, and their rationale is explained in Valdés Hernández et al. (2015). Formal written consent from all subjects and ethical approval was acquired from the Lothian Research Ethics Committee (09/S1101/54, LREC/2003/2/29, REC 09/81101/54), the NHS Lothian R + D Office (2009/W/NEU/14), and the Multi-Centre Research Ethics Committee for Scotland (MREC/01/0/56) and conducted according to the principles expressed in the Declaration of Helsinki.

**Table 1:** Summary of the acquisition and segmentation protocols present in the dataset.

Protocol	1	2	3
Number (test/train)	18/5	70/11	39/4
*T*_1_-w TR/TE/TI (ms)	9/440	9.7/3.984/500	
FLAIR TR/TE/TI (ms)	9002/147/2200	9000/140/2200	
Ground truth	Expert corrected histogram segmentation	Multispectral colour-fusion-based semi-automatic segmentation^a^	Expert corrected histogram segmentation
Lesion types present	WMH_pvo_	WMH/cortical infarcts	WMH_pvo_

a Valdés Hernández et al. Valdés Hernández et al., 2013a, Valdés Hernández et al., 2015.

All image sequences (from each patient) were co-registered using FSL-FLIRT (Jenkinson et al., 2002) and mapped to the patient's *T*_2_-w space. Lesions from images acquired under protocols 1 and 3 were extracted using histogram-based thresholding on FLAIR and manually rectified by an expert. Lesions from images acquired under protocol 2 (Table 1) were segmented by an expert following the procedure described in Valdés Hernández et al. Valdés Hernández et al., 2013a, Valdés Hernández et al., 2015, which uses a multispectral colour-fusion-based semi-automatic segmentation method and considers hyperintense signals that simultaneously appear in all *T*_2_-based sequences.

The 20 subjects with the lowest lesion volume (so as to maximise healthy tissue) were selected to form **T**^**train**^ and **F**^**train**^ and excluded from further analysis. The manual masks for these subjects were dilated by one voxel and used to mask out regions of pathology from the training process. Note that this step would not be necessary if pathology free subjects were available to form the training set.

### 3.2 Evaluation metrics

We computed a set of subject-wise similarity metrics to quantify the performance of each method by comparing segmentation volumes *V*_*a*_ to target volumes *V*_*t*_, and corresponding surfaces *S*_*a*_ and *S*_*t*_:

• *Dice Similarity Coefficient (DSC)*:
  A measure of overlap between the volume of the computed segmentations and the corresponding reference segmentations (Dice, 1945). Provides an overall measure of the accuracy of the computed segmentation, but becomes more sensitive to errors for small lesions. A DSC of 0 indicates no overlap, whilst a DSC of 1 indicates a perfect overlap.
  Defined as $\frac{2|V_{a}\cap V_{t}|}{\left|V_{a}|+|V_{t}\right|}$.
• *Average Symmetric Surface Distance (ASSD, mm)*: A measure of the average distances between the surface of the computed segmentations and the reference segmentations, and vice-versa. Provides an indication of how well the boundaries of the two segmentations align.
  where mindist(*p*,*S*) is the smallest Euclidean distance between surface point *p* and any point on *S*.
• *Hausdorff Distance (HD, mm)*: A measure of the maximal distance between the surfaces of the computed and reference segmentations. More sensitive to segmentation errors occurring away from segmentation boundaries than ASSD.
  Defined as max{{mindist(*a*,*S*_*t*_),*a* ∈ *S*_*a*_},{mindist (*t*,*S*_*a*_),*t* ∈ *S*_*t*_}}, where mindist(*p*,*S*) is the smallest Euclidean distance between point *p* and any point in *S* and max{*A*} returns the greatest value in set *A*.
• *Precision*: The proportion of the computed segmentation which overlaps with the reference segmentation. Provides an indication of over-segmentation. Ranges between 0 and 1.
  Defined as $\frac{\left|V_{a}\cap V_{t}\right|}{\left|V_{a}\right|}$.
• *Recall*: The proportion of the reference segmentation which overlaps with the computed segmentation. Provides an indication of under-segmentation. Ranges between 0 and 1.
  Defined as $\frac{\left|V_{a}\cap V_{t}\right|}{\left|V_{t}\right|}$.
  We also computed groupwise correlations across all test subjects:
• *Intra Class Correlation (ICC)*: A measure of correlation between |*V*_*t*_| and |*V*_*a*_|. Calculated as ICC(A,1) defined as in McGraw and Wong (1996). Scatter and Bland-Altman plots showing the relationship between these were also produced.
• *Correlation with Fazekas score*: Spearman's rank correlation coefficient calculated between |*V*_*a*_|/|*V*_*ic*_| and a combined Fazekas (Fazekas et al., 1987) score over all subjects, where |*V*_*ic*_| is a subject's intercranial volume mask. A Fazekas score is a clinical measure of WMH, comprising of two integers in the range [0,3] reflecting the degree of periventricular WMH and deep WMH respectively. For the purposes of this comparison the two scores were added giving a single value in the range [0,6].
• *Scatter and Bland-Altman plots*: Scatter and Bland-Altman plots showing the relationship between |*V*_*a*_|/|*V*_*ic*_| and |*V*_*t*_|/|*V*_*ic*_|. The lesion volumes were observed to be non-normal and hence non-parametric metrics were produced. The scatter plots allow us to see how closely the two sets of values are related, with a low variance distribution along the line *y* = *x* being desired. The Bland-Altman plots allow us a further measure of the agreement between the two sets of values, robust to sample selection (Bland and Altman, 2010). We plot $\frac{\left|V_{a}|/|V_{ic}|-|V_{t}|/|V_{ic}\right|}{0.5(|V_{a}|/|V_{ic}|+|V_{t}|/|V_{ic}|)}$ and desire the mean to be close to zero, indicating a lack of fixed bias, and variance to be small, indicating a high degree of agreement. Visually we also desire for there to be no trends or patterns in the data which would indicate a volume dependent bias.
  – Equation of best fit line: Of the form *y* = *mx* + *c*, found by minimising the sum of squared errors (SSE). Indicates how close the relationship between the two datasets is to the ideal (*y* = 1*x* + 0). A larger value of |*c*| indicates a constant error independent of lesion volume, whilst the a value of *x* differing from 1, indicates an error dependent on lesion volume.
  – *r*^2^: The square of the Pearson correlation coefficient. Indicates how strongly correlated the two volume measures are with a value of 1 indicating a perfect correlation.
  – SSE: Indicates how well the above equation fits the data.
  – RPC: Reproducibility coefficient. Indicates how well the automated method reproduces the results of the reference volumes.
  – CV: Coefficient of variation. Indicates the strength of agreement between the two volume measures.
  – Mean: Indicates a fixed bias if different from zero. P-values signaling this difference are also given.
  Finally, we computed two volume dependent metrics which provide additional insight into the conditions in which each method performs well, and where they are limited:
• *Lesion volume dependent DSC (DSC_*l*_)*: The DSC calculated within the bounding box of each lesion, separated into groups corresponding to very small (V*S* < 0.01 ml), small (0.01 ≤S < 0.1 ml), medium (0.1 ≤M < 1 ml), large (1 ≤L < 10 ml), very large (10 ml ≤VL) lesions. A lesion is defined as a single connected component within the reference segmentation. The bounding box of a lesion is defined as the smallest volume 3D box containing the lesion with dimensions parallel to the axes of the global coordinate system.
• *Subject volume dependent DSC (DSC_*s*_)*: The DSC for subjects separated into groups corresponding to very low ( <5 ml), low (5–10 ml), medium (10–15 ml) and high ( >15 ml) lesion volume according to reference segmentations.

### 3.3 Compared methods

• *LGA*: One of the methods available in the Lesion Segmentation Toolbox. LGA (Schmidt et al., 2012) is an unsupervised method which requires both a *T*_1_-w and a FLAIR image. The *T*_1_-w image is used to create a tissue type segmentation using an expectation maximisation approach. These tissue maps are propagated to the FLAIR image and used to create an initial lesion belief map which is binarised using a tunable threshold, *κ*. The authors suggest a *κ* value of 0.3, although they strongly encourage that this value be optimised for a particular dataset. The resulting segmentation is used as a seed for a region growing algorithm. The output of the algorithm is a probabilistic lesion map which must then be thresholded. Parameters (suggested): *κ* (0.3), threshold (0.5).
• *LPA*: The second algorithm available in the LST. LPA is a supervised algorithm which has been trained on 53 subjects with severe MS lesion patterns, and requires only a FLAIR image. A number of covariates for a logistic regression model are derived from the FLAIR image including a lesion belief map similar to the one produced by LST-LGA. The trained model in then used to assign a lesion probability estimate for each voxel, which is thresholded. Despite being supervised, the fact the model has been previously trained means it can be directly applied without requiring a training set. Parameters (suggested): threshold (0.5).
• *LesionTOADS* (Shiee et al., 2010): This unsupervised algorithm introduces lesion segmentation to a previously developed structural segmentation method - TOpology-preserving Anatomical Segmentation (TOADS) - by incorporating an additional lesion class. TOADS performs iterative segmentation driven by both statistical and topological atlases to ensure intensity and topological constraints are observed. LesionTOADS introduces a new class within the WM, with the union of the lesion and WM class following the same topological constraints as the original WM class. The algorithm requires both a *T*_1_-w and FLAIR image and outputs both a lesion and structural segmentation.

For each method, we performed experiments using both default parameters and optimised parameters based upon a grid search across one or two parameters which maximised DSC. For the proposed method these parameters relate to the CRF, with the default parameters being those suggested in the CRF implementation (available at^8^) adjusted for an isotropic voxel grid. During optimisation, two parameters were varied. *w*^(2)^ adjusts the relative weighting between the two CRF energy terms, and *σ*_*γ*_ determines how strongly homogeneity within the segmented region is enforced. Average subject-wise metrics and correlations for each method can be seen in Table 2, whilst volume dependent metrics for the optimal parameters can be seen in Table 4, Table 3. Significance testing at a 5% significance level was performed using paired Wilcoxon signed rank tests on subject wise metrics, and by comparing 95% confidence intervals for ICC.

**Table 2:** Table showing the results of each method over the whole dataset. Optimal parameter combinations indicated by *. Statistical differences between the closest competitor (optimised LPA) and the proposed method at a 5% significance level are in bold. For comparison, correlation between ground truth volumes and Fazekas scores is 0.829.

Method	Parameters	DSC	ASSD	HD	Prec.	Recall	ICC	Faz. corr.
LGA	*κ* = 0.3	0.382	5.77	48.6	0.925	0.265	0.693	0.782
	*t* = 0.5							
LPA	*t* = 0.5	0.536	2.60	37.3	0.926	0.416	0.874	0.846
LesionTOADS		0.497	2.74	34.3	0.667	0.498	0.488	0.358
LGA	*κ* = 0.11*	0.473	4.54	39.9	0.698	0.403	0.836	0.767
	*t* = 0.01*							
LPA	*t* = 0.15*	0.683	1.62	**33.3**	0.759	0.681	0.952	0.805
Proposed	*w*^(2)^ = 8*	**0.703**	**1.23**	38.6	0.763	0.695	**0.985**	0.862
	*σ*_*γ*_ = 2.5

**Table 3:** Lesion volume dependent DSC (DSC_*l*_) for each optimised method. Statistical differences between the closest competitor (optimised LPA) and the proposed method at a 5% significance level are in bold.

Method	<0.01 ml	0.01–0.1 ml	0.1–1 ml	1–10 ml	>10 ml
LesionTOADS	0.077	0.155	0.333	0.514	0.629
LGA	0.024	0.048	0.214	0.467	0.599
LPA	0.094	0.198	0.496	0.691	0.797
Proposed	**0.150**	**0.335**	**0.577**	**0.713**	0.807

**Table 4:** Subject volume dependent DSC (DSC_*s*_) for each optimised method. Whilst the proposed method obtains the largest DSC_*s*_ values, the differences with the closest competitor (optimised LPA) are not significant.

Method	<5 ml	5–10 ml	10–15 ml	>15 ml
LesionTOADS	0.157	0.440	0.426	0.614
LGA	0.343	0.334	0.374	0.577
LPA	0.558	0.615	0.569	0.762
Proposed	0.576	0.628	0.666	0.770

We were able to successfully run LPA and the proposed method on all subjects, however LGA and LesionTOADS failed to run on two and three subjects respectively. Intercranial volume was also unavailable for two subjects. Results are given across all subjects for which the method was successful, whereas comparisons between methods were only taken across subjects which were successfully processed across both methods.

We also analysed the results by grouping subjects into the three acquisition protocols and computing the average DSC over each protocol, giving further insight into the strengths and weaknesses of each method, Table 5.

**Table 5:** Table comparing average DSC for each method on images belonging to each protocol. Statistical differences between the closest competitor (optimised LPA) and the proposed method at a 5% significance level are in bold.

Protocol	1	2	3
LesionTOADS	0.431	0.535	0.445
LGA	0.322	0.453	0.568
LPA	0.688	0.678	0.690
Proposed	0.645	**0.710**	**0.719**

### 3.4 Clinical validation

In addition to the above quantitative evaluation, we also carry out a clinical validation by examining the coefficients of a general linear model formed from the normalised segmentation volumes of each method and a number of clinical and radiological variables. These coefficients are then compared to those formed from a model relating the variables to the reference segmentations. The models are composed as such:

(3) $$\begin{array}{lll} Vol{\%}_{i}^{method}= & \beta_{0}+\beta_{1}\;Age_{i}+\beta_{2}\;Gender_{i}+\beta_{3}\;Diabetes_{i} & \\ & +\beta_{4}\;Hypertension_{i}+\beta_{5}\;Hyperlipidaemia_{i}+\beta_{6}\;Smoking_{i} & \\ & +\beta_{7}\;Cholesterol_{i}+\beta_{9}\;PVSBG_{i}+\beta_{9}\;DeepAtrophy_{i}+\epsilon_{i}, \end{array}$$

where *Vol%*^*method*^ is the lesion segmentation volume for each method as a percentage of intercranial volume, *i* indicates a particular subject, *Diabetes*, *Hypertension* and *Hyperlipidaemia* are binary variables, *Smoking* is an integer (range [0,2] - never smoked, used to smoke, smokes), Cholesterol (mmol/L), *PV S*_*BG*_ is a radiological observation reflecting the number perivascular spaces in the basel ganglia (Potter et al., 2015), *DeepAtrophy* is a radiological observation reflecting the degree of deep cortical atrophy (Farrell et al., 2009), *ϵ* is a residual error term and *β* is the set of coefficients which minimises $\sum_{i}\epsilon_{i}$. *Gender* is included to remove bias but is not considered a risk factor and therefore not reported.

The strength of association between each clinical or radiological variable and the lesion volume produced by each method were measured by conducting a t-test for each coefficient *β*_*i*_ under the hypothesis that *β*_*i*_ = 0. By setting a 5% significance level, the set of variables which have the strongest association with the measured lesion volume was found for each method.

An additional set of models were formed by replacing *PV SBG*_*i*_ in Eq. (3) with *Fazekas*_*i*_, being the combined Fazekas score for subject *i*. Whilst expected to be strongly associated, comparing the *β*_9_ coefficient calculated for each automated method to that calculated for the reference segmentations provides a further indicator as to which methods more accurately model the process of producing the reference segmentations.

Note that evaluation is carried out across only the subjects (*n* = 96) for which all clinical and radiological variables are available.

## 4 Results and discussion

**Fig. 13:**
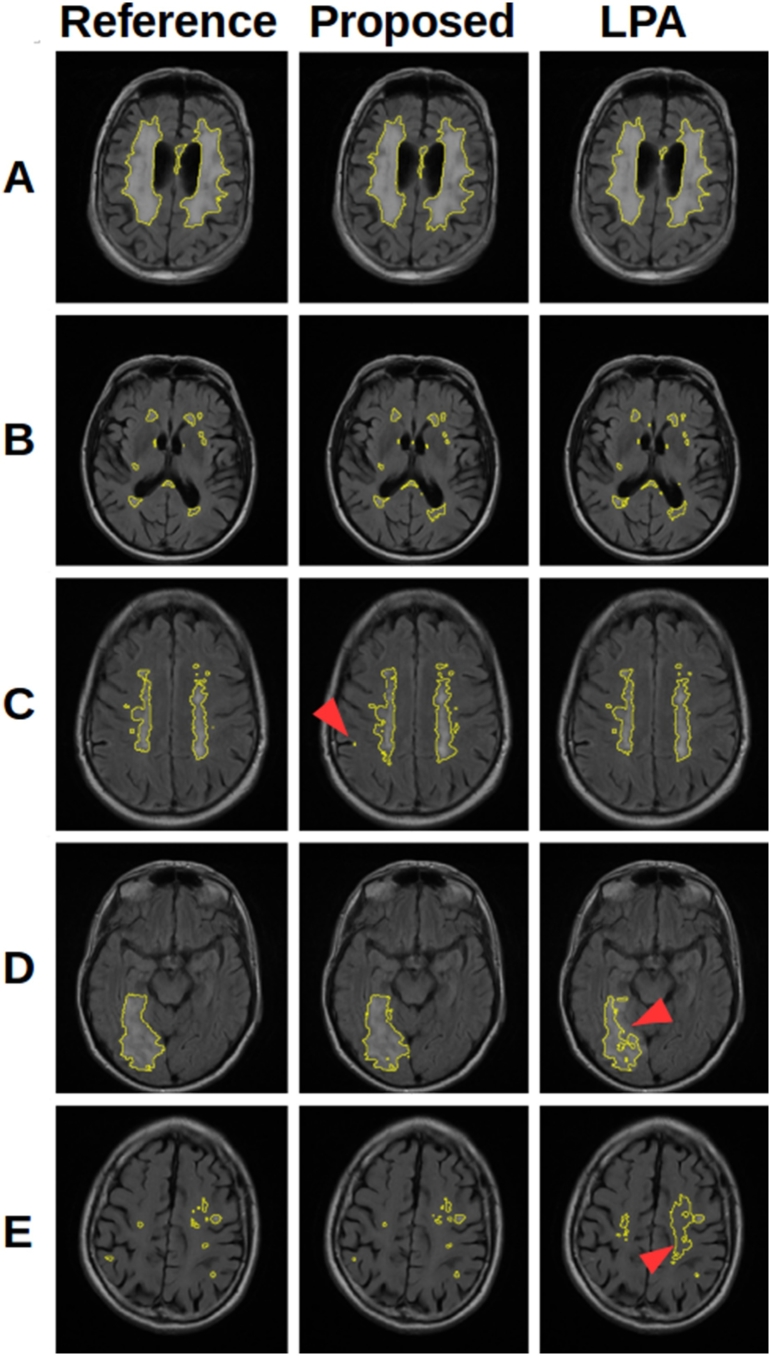
A selection of segmentations showing the features of the proposed method and LPA. (A) and (B) show cases where both methods perform well. (C) shows a case where the proposed method produces false positive voxels (arrow) in the GM, not present in LPA which does not consider GM. (D) shows a large infarct extending into the cortex where the extension into the cortex (arrow) is poorly segmented by LPA. (E) shows a case where small lesions are missed by LPA, despite considerable over segmentation (arrow).

When comparing methods it is necessary to understand the aims and limitations of each algorithm. The methods contained in the LST were developed to segment MS lesions, whilst LesionTOADS aims to segment both WMH and MS lesions. These methods are therefore only interested in lesions within the WM, and restrict their search to reflect this using a WM tissue segmentation. On the other hand, our proposed method aims to segment all hyperintense lesions on FLAIR, including WMH, MS lesions, and cortical infarcts, and as such, cannot restrict the search to the WM. Both approaches have advantages and disadvantages, which are reflected in the results and discussed in the following sections. The main advantage of restricting the search to the WM is that it avoids false positives occurring in the GM. This is important as GM can have a similar intensity distribution to WMH and MS lesions on FLAIR, and can therefore be a considerable source of false positives. The obvious drawback is that such methods will struggle to identify cortical infarcts. Fig. 13 shows some example segmentations demonstrating the consequences of these approaches.

### 4.1 Whole dataset analysis

When considering the dataset as a whole, Table 2 shows that the proposed method generally outperforms the existing methods, with significant improvements in DSC, ASSD and ICC. Despite being developed for and trained on MS lesions, LPA performs very well, and is the closest competitor across these metrics, with a significantly superior HD. This superior HD can be explained by the reduced likelihood of false positives in the GM when compared to the proposed method, as discussed earlier. Any tendency towards false positives far away from real lesions, such as in the GM, will be strongly punished by HD. LesionTOADS and LGA both fall well short of LPA and the proposed method. It is clear that the suggested thresholds of 0.5 and *κ* of 0.3 result in considerable under segmentation and overall poor results. It is however interesting to observe that these methods do achieve high correlations with Fazekas scores despite lower performance compared to ground truth segmentation. This suggests that a fully accurate segmentation may not be necessary to predict a Fazekas score. The proposed has the strongest correlation with Fazekas scores (0.862), which is stronger that of the reference segmentations (0.829), though with a P-value of 0.18, we cannot say conclusively that the automated method outperformed the reference segmentations in this regard. Similarly the power (56%, non-parametrically estimated through bootstrapping) of the DSC comparison between LPA and the proposed method suggests that additional data would help to strengthen our conclusions.

**Fig. 14:**
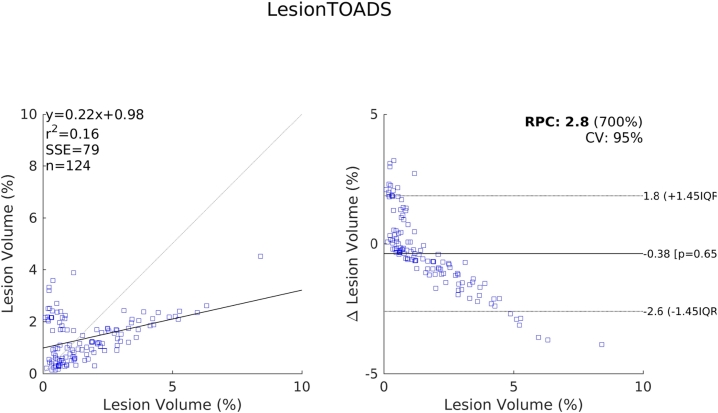
Scatter and Bland-Altman plots comparing of the lesion volumes (as a percentage of intercranial volume) from LesionTOADS to those from the reference segmentations.

**Fig. 15:**
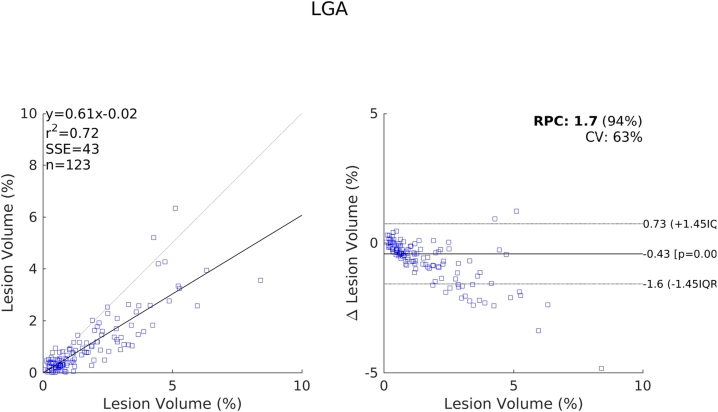
Scatter and Bland-Altman plots comparing of the lesion volumes (as a percentage of intercranial volume) from LGA to those from the reference segmentations.

**Fig. 16:**
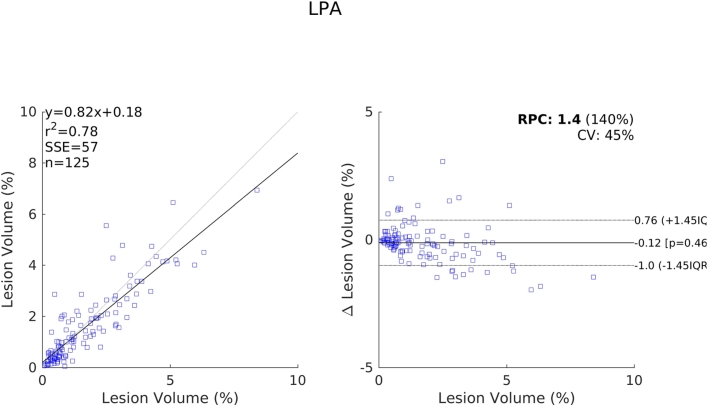
Scatter and Bland-Altman plots comparing of the lesion volumes (as a percentage of intercranial volume) from LPA to those from the reference segmentations.

**Fig. 17:**
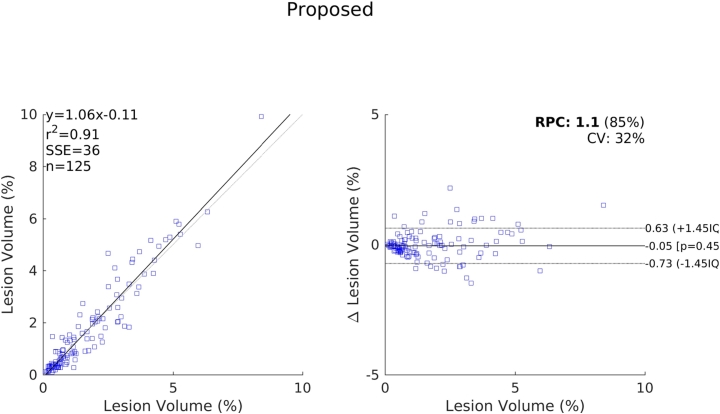
Scatter and Bland-Altman plots comparing of the lesion volumes (as a percentage of intercranial volume) from the proposed method to those from the reference segmentations.

The relative performance of each method compared to one another indicated by these results are further supported by the scatter and Bland-Altman plots shown in Fig. 14, Fig. 15, Fig. 16, Fig. 17. We see a clear visual improvement going from LesionTOADS to LGA, to LPA, and to the proposed method, along with an improvement in the associated metrics. A common feature of LesionTOADS, LPA and LGA is a tendency to underestimate lesion volumes at larger lesion loads, whilst the proposed method appears unaffected. One contributory factor towards this could be our intensity normalisation procedure which we chose so as to be unaffected by lesion load.

### 4.2 Volume specific analysis

When we divide the dataset into subsets with different lesion volumes we see that the proposed method performs better across all subsets. Whilst individually not significant at a 5% level due to the lower power of the subsets, the consistency of these result leads to the significantly higher DSC observed in Table 2. We also observe the trend that DSC increases as lesion volume increases, shown in Table 4. This is an expected result, and one which has been frequently observed (Griffanti et al., 2016). A similar trend is observed when examining results on individual lesions in Table 3. The smaller the lesion, the lower the expected DSC. This is a feature of DSC and can be explained by a number of factors. First, the larger lesions present in subjects with a high total volume of lesions have a higher ratio of internal to boundary voxels. Internal voxels tend to be more hyperintense and have more support from adjacent voxels, leading to easier segmentation. Secondly, smaller lesions tend to be less hyperintense, reducing the contrast with surrounding tissue, making them harder to segment. Finally if we assume a rate of false positives due to noise or artifacts independent of total lesion volume, these will have a much larger impact on the DSC for subjects with a low total lesion volume than those with a high total lesion volume where the potential for true positives to counter the effects of the false positives is much greater.

A consequence of the above is that the overall DSC reported in Table 2 is dominated by the ability of the algorithm to detect large lesions. Over 80% of the total volume of lesions belong to lesions with a size  >1 ml, and over 95% belong to lesions  >0.1 ml. However, small but strategically placed lesions can be clinically vital and the ability to detect these should form part of the evaluation of an algorithm. The results in Table 3 allow us to compare the performance of each method on differing sizes of lesion. We observe that whilst the proposed method and LPA get similar results on the larger lesions, the proposed method performs much better than the other methods at detecting smaller lesions.

### 4.3 Protocol specific analysis

It is possible to gain further insight into the merits of each method by looking at the results over each of the three protocols present in the dataset, allowing for more direct comparisons between the methods. It is important to remember that subdividing the dataset in this way leads to in a loss of sample power. Whilst the lower sample size is offset by stronger differences between LPA and the proposed method in the cases of protocols 2 and 3 (power = 74% and 57% respectively), these are still lower than desired and the small sample size for protocol 1 leads to a power of just 2%. As such the results should only be considered along with other factors, such as algorithm design, to lend support to hypotheses regarding the strengths and weaknesses of each method.

Images acquired under protocols 1 and 3 contain only WMHpvo and are therefore ideal cases for both LST methods and LesionTOADS due to the lack of cortical infarcts. On the other hand, images acquired under protocol 2 can contain both WMH and cortical infarcts, the latter being more likely to be segmented by the proposed method. The results in Table 5 suggest that LPA performs better on protocol 3 than on protocol 2, whilst the metrics for the proposed method are similar between the two protocols. This supports the hypothesis that LPA suffers in the presence of cortical infarcts.

Protocol 3 allows for a direct and fair comparison between the methods, as it does not contain cortical infarcts and is therefore not biased against the methods which only search in WM. Despite this, the proposed method significantly outperforms the other methods on protocol 3, indicating the superior results seen across the full dataset are not simply due to the ability to detect cortical infarcts.

However, both the proposed method and LGA perform worse on protocol 1 than protocols 2 and 3, whereas LPA performs equally well on protocols 1 and 3. Whilst the power of the comparison is extremely small, there are compelling reasons why LGA and the proposed method might not perform as well on protocol 1 as protocol 3. Whilst LPA uses only the subject's FLAIR image, LGA and the proposed method use both *T*_1_-w and FLAIR. The FLAIR acquisition protocol differs very little across the three protocols, however the *T*_1_-w acquisition does. The *T*_1_-w images acquired under protocol 1 come from a spoiled gradient echo sequence, as opposed to the magnetisation prepared fast gradient echo sequence used in protocols 2 and 3. This leads to lower contrast *T*_1_-w images in protocol 1, and a negative effect on the results of the two methods which use *T*_1_-w images.

Finally, recent work (Haller et al., 2016) has shown that protocol specific MR parameters can systematically bias the results of automated volume estimation of a number of brain structures by 4–5%. We must therefore consider the possibility of a similar effect could being present when estimating lesion volume. Whilst this is hard to observe from the results, given that the three protocols differ by more than just MR parameters, it should be considered as a potential contributory factor to explain the differences between the results from protocol 1 and those from 2 and 3.

### 4.4 Clinical validation

Looking for associations between clinical and radiological measurements and calculated lesion volumes provides an alternative way to compare methods. Whilst the dataset we use contains a variety of pathologies and degrees of abnormality, and as such we do not expect to find strong associations with all risk factors, comparing what associations are found to those found using the reference segmentations provides confirmation that the methods we are comparing produce segmentations with the same distribution across subjects.

Table 6 shows that there is a strong association between the reference segmentation volumes and perivascular spaces in the basel ganglia, deep atrophy and diabetes. This pattern is reflected in the results from LPA and the proposed method, suggesting good correspondence between these segmentations and the reference. The results from LGA agrees with two out of the three associations, but also suggests an association with cholesterol with is not present in the reference. The results from LesionTOADS find only an association with age, sharing no associations with that of the reference. These results are in keeping with our previous observations, reinforcing the belief that LPA and the proposed method both produce more accurate segmentations than the other two.

**Table 6:** P-values of the coefficients found using the model shown in Eq. (3). Bold indicates statistical significance of the coefficients from 0 at a 5% level.

WMH	Reference	LGA	LPA	Proposed	LesionTOADS
Age	0.82	0.88	0.11	0.55	**5** ×**10**^** −3**^
Diabetes	**0.03**	0.45	**0.01**	**0.02**	0.71
Hypertension	0.28	0.09	0.22	0.39	0.11
Hyperlipidaemia	0.37	0.87	0.24	0.29	0.78
Smoking	0.63	0.27	0.40	0.27	0.78
Cholesterol	0.95	**0.04**	0.12	0.11	0.53
PVSBG	**4** ×**10**^** −13**^	**7** ×**10**^** −7**^	**2** ×**10**^** −9**^	**2** ×**10**^** −8**^	0.13
DeepAtrophy	**0.02**	**2** ×**10**^** −5**^	**3** ×**10**^** −5**^	**6** ×**10**^** −4**^	0.40

The coefficients in Table 7 suggest that an increase in 1 in the combined Fazekas score is associated with an increase in reference lesion volume of 0.649. This association is most similar to that found from segmentations from the proposed method (0.717), with those from LPA (0.555) also similar. Again, LGA is next closest, followed by LesionTOADS.

**Table 7:** Coefficients found using the model shown in Eq. (3) with *Fazekas* in place of *PV SBG*. Bold indicates coefficients which are significantly different from 0 at a 5% level.

WMH	Reference	LGA	LPA	Proposed	LesionTOADS
Age	−3 ×10^ −4^	−5 ×10^ −4^	**0.019**	0.008	**0.032**
Diabetes	0.189	−0.083	**0.477**	0.248	−0.119
Hypertension	0.251	0.196	0.028	0.121	−0.118
Hyperlipidaemia	−0.098	−0.065	−0.029	0.043	−0.103
Smoking	−0.030	0.029	0.014	0.033	0.003
Cholesterol	0.107	−0.026	0.017	0.028	0.077
Fazekas	**0.649**	**0.320**	**0.555**	**0.717**	**0.150**
DeepAtrophy	**0.002**	**0.012**	**0.013**	**0.010**	− **0.002**

## 5 Conclusion

We have presented a method for brain lesion segmentation through the use of an image synthesis algorithm, regardless of underlying pathology. We have shown that an apparently healthy FLAIR image can be synthesised from a subject's *T*_1_-w image, and that the differences between this synthetic FLAIR and the real FLAIR can be combined with information from the real FLAIR to indicate the location of lesions. The resulting segmentations are objectively superior when compared to a ground truth to a number of established methods across a range of clinically relevant metrics, including a particularly strong ability to detect smaller lesions. The results allow us to make the following conclusions:

• The proposed method significantly outperforms the existing methods on a heterogeneous dataset across most metrics.
• The proposed method does particularly well in cases with cortical infarcts, which are undetected by other methods.
• One of the biggest advantages of the proposed method is its ability to detect smaller lesions, something which, depending on the application, could be clinically highly relevant.
• Whilst not catastrophic, a limitation of the proposed method is that it requires both FLAIR and *T*_1_-w images, and any significant changes in *T*_1_-w acquisition protocols may negatively impact performance, see Table 5.

Future work will involve extending the framework to allow for the detection of unexpected hypointensities, such as lacunar cavities, and other hallmarks of SVD such as microbleeds and enlarged perivascular spaces. Modifying the approach to more readily handle a variety of acquisition protocols will also make the method more robust. This could be achieved though an extension of the regression model itself, or as a preprocessing step using sequence normalisation (Roy et al., 2013) which could also provide improved intensity normalisation.

## Footnotes

1 www.statistical-modelling.de/lst.

2 www.biomedia.doc.ic.ac.uk/software/mirtk/.

3 www.bic.mni.mcgill.ca/ServicesAtlases/ICBM152NLin2009.

4 www.doc.ic.ac.uk/~cl6311/software.

5 www.applied-statistics.de/lst.

6 www.nitrc.org/projects/toads-cruise.

7 www.sbirc.ed.ac.uk.

8 github.com/Kamnitsask/dense3dCrf.
